# Changes in Life Expectancy After Stroke Among Americans Aged 50 and Above by Sex, 1996–2018

**DOI:** 10.1111/jgs.70585

**Published:** 2026-07-11

**Authors:** Octavio Bramajo, Moumita Chakraborty, Lynda Lisabeth, Neil Mehta

**Affiliations:** ^1^ Antrophometrics and Historical Epidemiology Group, Institute for Evolutionary Medicine University of Zurich Zurich Switzerland; ^2^ Department of Biostatistics & Data Science University of Texas Medical Branch Galveston Texas USA; ^3^ School of Public Health University of Michigan Ann Arbor Michigan USA; ^4^ Department of Epidemiology and Population Health University of Texas Medical Branch Galveston Texas USA

**Keywords:** cardiovascular health, life expectancy, multistate models, stroke, United States

## Abstract

**Background:**

Stroke remains a leading cause of mortality and disability in the United States. As the population ages, understanding trends in survival among stroke survivors is critical for healthcare planning and evaluating secondary prevention efforts.

**Methods:**

Using longitudinal data from the nationally representative Health and Retirement Study (1996–2018; 39,084 individuals, 225,316 person‐wave observations), in which stroke status is self‐reported, we computed life expectancy at Age 50 (LE50) for those with and without a previous stroke history using discrete‐time multistate models (a Markov chain approach for panel data). Estimates were stratified by sex and compared across two 10‐year periods (1996–2006 vs. 2008–2018), with 95% confidence intervals obtained from 1000 bootstrap iterations. Cox proportional hazards models with progressively expanded covariate sets (sociodemographics, heart disease, BMI, insurance type, race/ethnicity) were fitted as exploratory analyses to assess the robustness of stroke–mortality associations to potential confounding.

**Results:**

LE50 among individuals with a self‐reported previous stroke history at baseline increased by 3.1 years (95% CI: 1.7–4.4) between 1996–2006 and 2008–2018 for both sexes combined, with men gaining 3.4 years (2.0–4.8) and women 2.5 years (0.8–4.3). Gains exceeded those in the general population (1.1 years, 0.6–1.6). Point estimates suggested somewhat larger gains among men, though confidence intervals overlapped and a statistically significant sex difference could not be confirmed. For first‐time incident strokes during the observation period, post‐stroke survival showed no statistically significant change across periods.

**Conclusions:**

Long‐term consequences of stroke appear to be becoming less lethal, likely reflecting improvements in secondary prevention and post‐stroke long‐term care. Nonetheless, a gap of approximately 10 years in LE50 between stroke survivors and the general population persisted in 2008–2018. Continued investment in both acute stroke care and long‐term secondary prevention remains essential to further close this gap.

## Summary

- Key points
  ○ Question: How has life expectancy after stroke changed in the United States over the past two decades among Americans aged 50 and above, and do trends differ by sex?
  ○ Findings: Life expectancy at Age 50 among stroke survivors increased by 3.1 years between 1996–2006 and 2008–2018, with gains exceeding those in the general population (approximately 1 year). Point estimates suggested somewhat larger gains among men (approximately 3.4 years) than women (approximately 2.5 years), though confidence intervals overlapped substantially. Survival after incident (first‐time) strokes showed minimal improvement. These findings were robust to expanded confounding control including heart disease, BMI, insurance type, and race/ethnicity.
  ○ Meaning: Improved long‐term survival among stroke survivors suggests effective secondary prevention and post‐stroke care. While point estimates suggest differential gains by sex, further research is needed to confirm whether these patterns reflect true sex disparities in post‐stroke outcomes.
- Why does this paper matter?
  ○ This study provides the first national estimates of life expectancy trends among stroke survivors in over two decades at a population level in a multistate framework, revealing substantial improvements in long‐term stroke survival that outpace general population gain, but still represent a significant shortfall of lifespans. A series of sensitivity analyses further support the validity of the results.

## Graphical abstract

Life expectancy at Age 50 improved more for individuals with prior stroke history (near 3 years) than for the general population (near 1 year) between 1996–2006 and 2008–2018. Gains were larger among males than females. A substantial survival gap between stroke survivors and the general population persists.

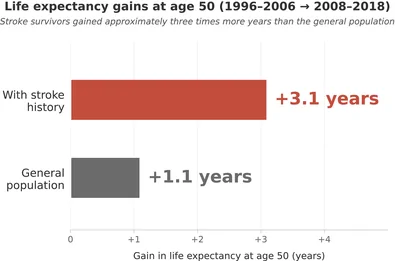

## 1 Introduction

Stroke remains a leading cause of mortality and disability in the United States, ranking as the fifth leading cause of death, with approximately 795,000 Americans experiencing a stroke annually [1]. Despite significant advances in prevention and treatment over recent decades [2, 3], stroke continues to impose a substantial burden on population health, healthcare systems, and families [4, 5]. Recent analyses have also revealed concerning trends in stroke mortality in the United States and other high‐income countries. While the period from 1990 to 2009 witnessed substantial declines in CVD mortality [6, 7], including stroke mortality [5, 8, 9], the 2010s saw a marked stagnation in stroke mortality trends [10, 11, 12]. The stagnation in stroke mortality parallels broader patterns of CVD mortality stagnation [13, 14, 15].

Trends in stroke mortality are a function of both trends in the incidence of stroke and trends in survival after stroke. In this paper, we focus on the latter, examining changes in survival and life expectancy among those who have experienced a stroke, which can provide critical insights into health system successes in treating stroke and managing post‐stroke outcomes. There have been few recent US studies that have examined changes in long‐term survival after stroke, although evidence from other high‐income countries indicate improvements in survival for ischemic strokes since the 1990s [16, 17, 18]. A recent study on a US midwestern population found that the 5‐year risk of death after an acute ischemic stroke declined modestly, from 53% to 48%, between 1993/94 and 2015 [17], while another study found that the use of specific mechanical interventions might be associated with a decline in mortality after 90 days of having an acute ischemic stroke in the last decade [19].

Our objective is to investigate changes in (healthy) life expectancy at Age 50 (comparing individuals with and without stroke history) in a nationally representative US sample with a set of discrete‐time multistate models. We use data from the Health and Retirement Study (HRS) and examine changes between 1996–2006 and 2008–2018. We investigated differences by sex as men typically face higher age‐adjusted stroke incidence than women, though women's longer life expectancy results in a higher lifetime stroke risk and greater absolute numbers of stroke deaths [4].

## 2 Data and Methods

The HRS is a longitudinal, comprehensive, national representative survey of Americans aged 50+ collecting data on health, health‐related behaviors, and socio‐demographic factors. It began in 1992. The National Institute on Aging (grant number NIA U01AG009740) sponsors the HRS at the University of Michigan. We used the RAND HRS longitudinal file that provides a harmonized set of variables across waves [20]. The HRS uses a steady‐state sampling design in which a new nationally representative sample of adults aged approximately 51–56 is enrolled every 6 years, maintaining cross‐sectional representativeness of Americans aged 50 and over at each wave [21]. As this study was a secondary analysis of existing deidentified publicly available data, IRB approval was not required under 45 CFR §46.101(b)(4). The study followed the Strengthening the Reporting of Observational Studies in Epidemiology (STROBE) reporting guideline [22].

We stratified our analysis into two 10‐year periods, considering stroke events between 1996 and 2006 (HRS waves 3–8) and between 2008 and 2018 (HRS waves 9–14). We excluded the 2020 and 2022 waves because the pandemic created substantial disruptions to mortality patterns and healthcare delivery. Stroke (and all other health measures) is identified from a question asking about stroke history (“Has a doctor ever told you that you had a stroke?”). While this may introduce ascertainment bias, stroke has relatively unambiguous clinical manifestations, and prior validation studies have shown reasonable concordance between self‐reported and medically verified stroke [23, 24]. Throughout this analysis, baseline stroke or previous stroke history refers to a self‐reported history of stroke present at the first interview within each observation period (1996–2006 or 2008–2018), regardless of when the original stroke occurred. The LE50 estimate for individuals with baseline stroke history therefore represents the expected remaining years for a hypothetical 50‐year‐old in the stroke‐history state, derived from age‐specific transition probabilities observed across the full age range during the period (as a reference, 14.6% of the strokes at baseline were reported by individuals under Age 55).

We defined incident strokes as a report of stroke in any subsequent wave within a period conditional on a report of no previous stroke in the first period. We included individuals between 50 and 108 years of age at the time of interview.

As an exploratory measure to observe which additional factors are associated with mortality risk and stroke history, we estimated a series of Cox proportional hazard models predicting mortality using an analytical sample that pooled data from both periods, implemented with the survival package in R software [25]. We estimated sex‐specific models (to avoid any possible violations of the proportionality hazard assumptions in the sex variable). Model 1 included age, an indicator variable for the 2008–2018 period, previous stroke history at baseline, and an interaction between previous stroke and period. Model 2 added incident stroke (if the respondent experienced a stroke during the observation period) and its period interaction. Age and incident stroke were treated as time‐varying covariates. Model 3 controlled for educational attainment, additional demographics (race/ethnicity and health insurance type) and health risk factors. Educational attainment was categorized as high (college and above), medium (high school graduate or some college), or low (less than high school). Health risk factors included smoking history (ever smoked), diabetes history (ever diagnosed), hypertension history (ever diagnosed with high blood pressure), heart diseases (ever diagnosed with heart problems). BMI (categorized as underweight, normal, overweight, or obese), health insurance type (private only, government only, both, or uninsured), and race/ethnicity (Non‐Hispanic White, Non‐Hispanic Black, Hispanic, or Non‐Hispanic Other). Model 4 added period interactions for these additional covariates. The proportional hazards assumption was evaluated using Schoenfeld residuals. Given that our subsequent analytical strategy involves discrete‐time multistate models stratified by sex and period based on fitted multinomial logistic regressions [26], any possible non‐proportionality of period effects is accommodated by design (since multistate models do not need this assumption for life expectancy estimations), limiting Cox models to exploratory purposes between covariates. Additionally, because the HRS does not establish the timing of previous strokes for those with stroke history at baseline, proportional hazards models are appropriate to assess average hazards across both 10‐year periods. Similar Cox regression approaches have been used in prior stroke survival studies [27, 28, 29].

We estimated life expectancy at Age 50 (LE50) using discrete‐time multistate models (DTMS), implemented with the dtms package in R [26, 30]. LE50 represents the average number of additional years a person is expected to live beyond Age 50 under the mortality conditions of the observed period. It is a population‐level summary measure: it does not predict outcomes for any specific person, but instead summarizes the survival prospects of a hypothetical cohort exposed to the age‐specific transition probabilities observed during the period [31]. In our framework, LE50 is decomposed into the expected years lived stroke‐free and the expected years lived with stroke history.

The discrete‐time framework is appropriate for the HRS because data are collected at fixed 2‐year intervals; unlike continuous‐time multistate models, which estimate instantaneous transition rates and require assumptions about what occurs between observation points, the discrete‐time approach directly models transition probabilities at each observed interval. Because age is expressed in 2‐year intervals (meaning that we follow survival for 10 years), the years 2007 and 2019 fall between waves and are not directly captured in either period. Transitions between states were based on incident stroke reports between interviews (from someone with no previous stroke history to stroke), with death as an absorbing state (Figure S1). The dtms_fit function (in the dtms package) fits a series of multinomial logistic regressions to inform the transition risk from each state to death (as an absorbing state) and across non‐death states. We stratified state‐specific life expectancy by sex and by 10‐year period. We used the bootstrap feature in the R package dtms_boot to estimate 95% confidence intervals (CIs) associated with each stroke‐history state, with 1000 iterations.

A total of 17,334 individuals contributed observations to both the 1996–2006 and 2008–2018 periods. This overlap is inherent to the longitudinal design of the HRS and does not constitute double‐counting (since they enter each period in a potentially different state, at a different age, with different covariates, reflecting the HRS refreshment design, as Tables S1 and S2 show). The discrete‐time multistate models estimate transition probabilities conditional on an individual's health state at each time point under a Markov assumption; that is, future transitions depend only on current state and age, not on prior history. An individual who was stroke‐free in 1996–2006 but experienced a stroke before 2008 enters the second period in the “Stroke History” state, contributing appropriately different transition information to each period. Prior multistate life expectancy analyses with refreshment samples have used the same partitioning approach [32, 33, 34, 35].

The analytical sample comprised 39,084 unique individuals (17,209 males, 21,875 females) contributing 225,316 person‐wave observations. No individuals with missing sex were reported. Missingness at the observation level was low: stroke status was missing in 90 observations (0.04%), education in 41 (0.02%), and smoking history in 1686 (0.75%). Hypertension, diabetes, period, age, and insurance type had no missing values. The base models (Models 1–2) used 223,518 complete observations. The expanded models (Models 3–4), which additionally required heart disease, BMI, insurance type, and race/ethnicity, used 219,891 complete observations. Given the low share of missingness, complete‐case analysis was used throughout. See Material S1 for a complete definition of variables used for this study and Table S3 for a detailed missing data report.

## 3 Results

Table 1 presents descriptive characteristics at baseline by period and sex. Approximately 57% of the sample was female in both periods. Mean age was similar across groups, ranging from 63.3 to 64.2 years. The prevalence of stroke history at baseline was higher among males (6.3% in 1996–2006, 6.9% in 2008–2018) than females (4.9% and 5.8%, respectively). Mean BMI increased from 27.1 to 28.6 in males and from 26.8 to 28.7 in females. Total deaths were 7040 in 1996–2006 and 6749 in 2008–2018, and 17,334 individuals appeared in both periods.

**TABLE 1:** Descriptive characteristics at baseline, by period.

	1996–2006		2008–2018	
	Males (*n* = 11,887)	Females (*n* = 15,566)	Males (*n* = 12,481)	Females (*n* = 16,484)
Age, mean (SD)	63.9 (10.3)	64.2 (11.5)	63.3 (11.0)	63.7 (11.8)
Stroke at baseline (%)	744 (6.3%)	768 (4.9%)	855 (6.9%)	950 (5.8%)
Ever smoked (%)	8529 (72.6%)	7357 (47.6%)	8079 (65.1%)	8075 (49.2%)
Hypertension (%)	4733 (39.8%)	6436 (41.3%)	6727 (53.9%)	8911 (54.1%)
Diabetes (%)	1646 (13.8%)	1803 (11.6%)	2732 (21.9%)	3188 (19.3%)
Other heart disease (%)	2767 (23.3%)	2853 (18.3%)	2928 (23.5%)	3097 (18.8%)
BMI, mean (SD)	27.1 (4.5)	26.8 (5.9)	28.6 (5.4)	28.7 (6.8)
Education				
Low	3855 (32.4%)	4947 (31.8%)	3225 (25.8%)	4047 (24.6%)
Medium	5436 (45.8%)	8301 (53.3%)	6186 (49.6%)	9140 (55.5%)
High	2589 (21.8%)	2313 (14.9%)	3066 (24.6%)	3296 (20.0%)
Insurance				
Private only	5143 (43.3%)	6570 (42.2%)	4463 (35.8%)	5951 (36.1%)
Government only	2343 (19.7%)	3357 (21.6%)	3740 (30.0%)	5132 (31.1%)
Both	3466 (29.2%)	4268 (27.4%)	2713 (21.7%)	3593 (21.8%)
Uninsured	935 (7.9%)	1371 (8.8%)	1565 (12.5%)	1808 (11.0%)
Race/ethnicity				
Non‐Hispanic White	9023 (76.0%)	11,455 (73.6%)	7797 (62.5%)	10,017 (60.8%)
Non‐Hispanic Black	1568 (13.2%)	2432 (15.6%)	2312 (18.5%)	3477 (21.1%)
Hispanic	1008 (8.5%)	1336 (8.6%)	1801 (14.4%)	2314 (14.0%)
Non‐Hispanic Other	279 (2.3%)	340 (2.2%)	558 (4.5%)	671 (4.1%)

*Note:* Baseline defined as first observation per individual per period. Person‐years: 1996–2006 = 1,088,586; 2008–2018 = 1,109,014. Total deaths: 1996–2006 = 7040; 2008–2018 = 6749. 17,334 individuals appeared in both periods.

Tables 2 and 3 present hazard ratios from the exploratory sex‐specific Cox proportional hazards models examining all‐cause mortality risk (with stroke status across two periods for males and females as a covariate), respectively. All estimates are hazard ratios corresponding to reference categories (with 95% CIs in parentheses). We present results from all models to demonstrate the robustness of findings across different model specifications. Model fit improved substantially from the unadjusted to fully adjusted specifications (C‐statistic: 0.760–0.793 for males and 0.789–0.819 for females; AIC decreased from 141,832 to 130,238 for males and from 162,686 to 150,859 for females).

**TABLE 2:** Cox proportional hazards models—Males.

Variable	Model 1	Model 2	Model 3	Model 4
*N* (observations)	94,615	85,547	84,269	84,269
Events (deaths)	6477	6218	6078	6078
C‐statistic	0.760	0.758	0.793	0.793
AIC	141,832	135,034	130,238	130,234
Nagelkerke *R* ^2^	0.079	0.080	0.102	0.102
Main effects				
Baseline stroke	2.2 (2.02–2.47)***	2.13 (1.92–2.4)***	1.67 (1.51–1.86)***	1.69 (1.52–1.88)***
Incident stroke	—	1.84 (1.58–2.1)***	1.55 (1.32–1.81)***	1.55 (1.33–1.82)***
Period 2008–2018	0.87 (0.83–0.92)***	0.88 (0.83–0.93)***	0.90 (0.85–0.95)***	0.95 (0.72–1.24)
Age (per year)	1.09***	1.08***	1.07***	1.07***
Education: Medium	—	—	0.83 (0.78–0.88)***	0.91 (0.84–0.98)*
Education: High	—	—	0.60 (0.55–0.65)***	0.64 (0.57–0.72)***
Ever smoked	—	—	1.38 (1.29–1.46)***	1.41 (1.29–1.54)***
Hypertension	—	—	1.20 (1.14–1.27)***	1.15 (1.07–1.23)***
Diabetes	—	—	1.53 (1.44–1.62)***	1.59 (1.47–1.73)***
Heart disease	—	—	1.41 (1.34–1.49)***	1.41 (1.31–1.52)***
BMI: Underweight (UW)	—	—	2.72 (2.40–3.09)***	3.0 (2.51–3.47)***
BMI: Overweight (OW)	—	—	0.60 (0.57–0.64)***	0.61 (0.56–0.66)***
BMI: Obese (OB)	—	—	0.50 (0.47–0.54)***	0.49 (0.44–0.55)***
Insurance: Government	—	—	1.62 (1.44–1.83)***	1.56 (1.34–1.81)***
Insurance: Both	—	—	1.51 (1.34–1.70)***	1.43 (1.23–1.66)***
Insurance: Uninsured	—	—	1.66 (1.39–1.98)***	1.47 (1.14–1.90)**
Race/ethnicity: NH‐Black	—	—	1.07 (0.99–1.15)	1.17 (1.05–1.29)**
Race/ethnicity: Hispanic	—	—	0.79 (0.71–0.87)***	0.94 (0.81–1.09)
Race/ethnicity: NH‐Other	—	—	0.89 (0.74–1.08)	1.09 (0.84–1.42)
Stroke * period interactions				
Baseline stroke * period	0.95 (0.83–1.10)	0.97 (0.84–1.12)	1.03 (0.89–1.19)	1.00 (0.86–1.16)
Incident stroke * period	—	0.93 (0.74–1.17)	1.00 (0.79–1.26)	0.99 (0.78–1.25)
Other period interactions				
Smoking * period	—	—	—	0.96 (0.85–1.08)
Education: Medium * period	—	—	—	0.82 (0.74–0.93)**
Education: High * period	—	—	—	0.86 (0.73–1.00)
Hypertension * period	—	—	—	1.11 (0.99–1.24)
Diabetes * period	—	—	—	0.92 (0.82–1.03)
Heart disease * period	—	—	—	1.00 (0.90–1.11)
BMI underweight * period	—	—	—	0.84 (0.65–1.08)
BMI overweight * period	—	—	—	0.99 (0.88–1.11)
BMI obese * period	—	—	—	1.04 (0.89–1.21)
Insurance: Government * period	—	—	—	1.11 (0.88–1.39)
Insurance: Both * period	—	—	—	1.15 (0.92–1.45)
Insurance: Uninsured * period	—	—	—	1.30 (0.91–1.87)
Race/ethnicity: NH‐Black * period	—	—	—	0.85 (0.73–0.98)*
Race/ethnicity: Hispanic * period	—	—	—	0.72 (0.58–0.88)**
Race/ethnicity: NH‐Other * period	—	—	—	0.68 (0.47–0.99)*

*Note:* Hazard ratios (95% CI). Reference: No stroke, period 1996–2006, Age 50, low education, never smoked, no HBP, no diabetes, normal BMI, private insurance, Non‐Hispanic White. Dash (—) = variable not in model.

*** *p* < 0.001.

** *p* < 0.01.

* *p* < 0.05.

**TABLE 3:** Cox proportional hazards models—Females.

Variable	Model 1	Model 2	Model 3	Model 4
*N* (observations)	130,611	120,903	117,496	117,496
Events (deaths)	7299	7141	6912	6912
C‐statistic	0.789	0.787	0.819	0.820
AIC	162,686	158,192	150,859	150,834
Nagelkerke *R*²	0.090	0.091	0.111	0.111
Main effects				
Baseline stroke	2.34 (2.12–2.6)***	2.2 (2.00–2.5)***	1.82 (1.64–2.02)***	1.82 (1.64–2.02)***
Incident stroke	—	2.12 (1.86–2.42)***	1.86 (1.63–2.12)***	1.84 (1.61–2.10)***
Period 2008–2018	0.96 (0.91–1.00)	0.96 (0.91–1.01)	0.95 (0.90–1.00)*	0.85 (0.65–1.10)
Age (per year)	1.09***	1.09***	1.08***	1.08***
Education: Medium	—	—	0.77 (0.73–0.81)***	0.76 (0.71–0.82)***
Education: High	—	—	0.63 (0.57–0.68)***	0.68 (0.60–0.78)***
Ever smoked	—	—	1.37 (1.31–1.44)***	1.29 (1.20–1.38)***
Hypertension	—	—	1.30 (1.23–1.37)***	1.31 (1.22–1.41)***
Diabetes	—	—	1.64 (1.55–1.73)***	1.97 (1.81–2.1)***
Heart disease	—	—	1.48 (1.40–1.55)***	1.40 (1.30–1.50)***
BMI: Underweight (UW)	—	—	2.34 (2.16–2.53)***	2.40 (2.16–2.66)***
BMI: Overweight (OW)	—	—	0.69 (0.65–0.74)***	0.64 (0.59–0.70)***
BMI: Obese (OB)	—	—	0.64 (0.60–0.69)***	0.60 (0.54–0.67)***
Insurance: Government	—	—	1.70 (1.49–1.92)***	1.63 (1.38–1.92)***
Insurance: Both	—	—	1.46 (1.28–1.65)***	1.38 (1.18–1.62)***
Insurance: Uninsured	—	—	1.97 (1.64–2.35)***	1.93 (1.50–2.48)***
Race/ethnicity: NH‐Black	—	—	0.99 (0.93–1.06)	1.02 (0.92–1.12)
Race/ethnicity: Hispanic	—	—	0.65 (0.59–0.72)***	0.69 (0.59–0.80)***
Race/ethnicity: NH‐Other	—	—	0.82 (0.68–0.99)*	0.93 (0.70–1.24)
Stroke * period interactions				
Baseline stroke * period	0.94 (0.82–1.08)	0.96 (0.84–1.11)	0.93 (0.80–1.07)	0.93 (0.80–1.07)
Incident stroke * period	—	0.95 (0.79–1.16)	0.96 (0.79–1.16)	0.97 (0.80–1.18)
Other period interactions				
Smoking * period	—	—	—	1.13 (1.03–1.25)*
Education: Medium * period	—	—	—	1.02 (0.92–1.14)
Education: High * period	—	—	—	0.87 (0.73–1.03)
Hypertension * period	—	—	—	0.97 (0.87–1.09)
Diabetes * period	—	—	—	0.72 (0.64–0.81)***
Heart disease * period	—	—	—	1.11 (1.00–1.22)*
BMI underweight * period	—	—	—	0.96 (0.82–1.13)
BMI overweight * period	—	—	—	1.15 (1.02–1.30)*
BMI obese * period	—	—	—	1.12 (0.98–1.29)
Insurance: Government * period	—	—	—	1.08 (0.85–1.37)
Insurance: Both * period	—	—	—	1.11 (0.87–1.41)
Insurance: Uninsured * period	—	—	—	1.05 (0.73–1.50)
Race/ethnicity: NH‐Black * period	—	—	—	0.96 (0.83–1.09)
Race/ethnicity: Hispanic * period	—	—	—	0.92 (0.75–1.13)
Race/ethnicity: NH‐Other * period	—	—	—	0.80 (0.55–1.17)

*Note:* Models 1–2 use age‐timescale with stroke and period. Model 3 adds education, smoking, hypertension, diabetes, heart disease, BMI, insurance, race/ethnicity. Model 4 adds covariate * period interactions. Hazard ratios (95% CI). Reference: No stroke, period 1996‐2006, age 50, low education, never smoked, no HBP, no diabetes, normal BMI, private insurance, Non‐Hispanic White. Dash (—) = variable not in model.

*** *p* < 0.001.

* *p* < 0.05.

Baseline stroke history was associated with substantially elevated mortality risk in both sexes. Despite attenuation from the coefficients from model 1, in the fully adjusted Model 3 the hazard ratio was 1.67 (95% CI: 1.51–1.86) higher for males and 1.82 (95% CI: 1.64–2.02) for females compared to those individuals without a previous baseline stroke. Incident stroke during follow‐up conferred similarly elevated risk, with males having a hazard ratio of 1.55 (95% CI: 1.32–1.81) and females of 1.86 (95% CI: 1.63–2.12). Point estimates for both stroke variables were somewhat higher for females than for males (though CIs overlapped).

Period interactions (for 2008–2018) for both baseline stroke and incident stroke were not statistically significant in either sex across all model specifications. These results indicate that increased mortality risk associated with stroke did not change significantly between periods beyond changes captured by the general population trend. Model 4 period interactions revealed that the elevated mortality risk associated with diabetes diminished between periods, particularly among females (interaction HR = 0.72, *p* < 0.001), likely reflecting improvements in diabetes management. Smoking showed worsening relative mortality among females (interaction HR = 1.13, *p* < 0.05). Among males, medium education and Hispanic ethnicity showed improving relative survival over time.

Other covariates behaved as expected. Higher education, overweight, and obese BMI categories were protective, while smoking, diabetes, hypertension, heart disease, underweight BMI, government‐only or no insurance, and being underweight were associated with increased mortality risk. The protective association of overweight and obesity with mortality is consistent with the well‐documented obesity paradox in older adults [36]. After controlling for age, Hispanic ethnicity was associated with lower mortality compared to non‐Hispanic Whites, consistent with the Hispanic paradox [37, 38]. This association was not a product of age distribution, since a lower Hispanic mortality was found across‐all age groups, as Table S4 indicates (and Table S5 presents the mean age by race‐ethnicity group).

Schoenfeld residual tests (Table S6) indicated proportional hazards violations for the period variable, which is expected given that it captures the secular trends of mortality change over a decade in the partitioned dataset. The baseline stroke, incident stroke, and their period interactions which are the main exposure variables for the subsequent DTMS models satisfied the proportional hazards assumption across all model specifications (all *p* > 0.05). Among females, heart disease, BMI category, insurance type, and race/ethnicity showed PH violations in Models 3 and 4, indicating time‐varying covariate effects. These violations do not affect the stroke coefficients, which satisfy the proportional hazards assumption independently. Moreover, the DTMS models, which provide the primary life expectancy estimates, do not need to assume proportional hazards, leaving the Cox models associations as exploratory.

**FIGURE 1:**
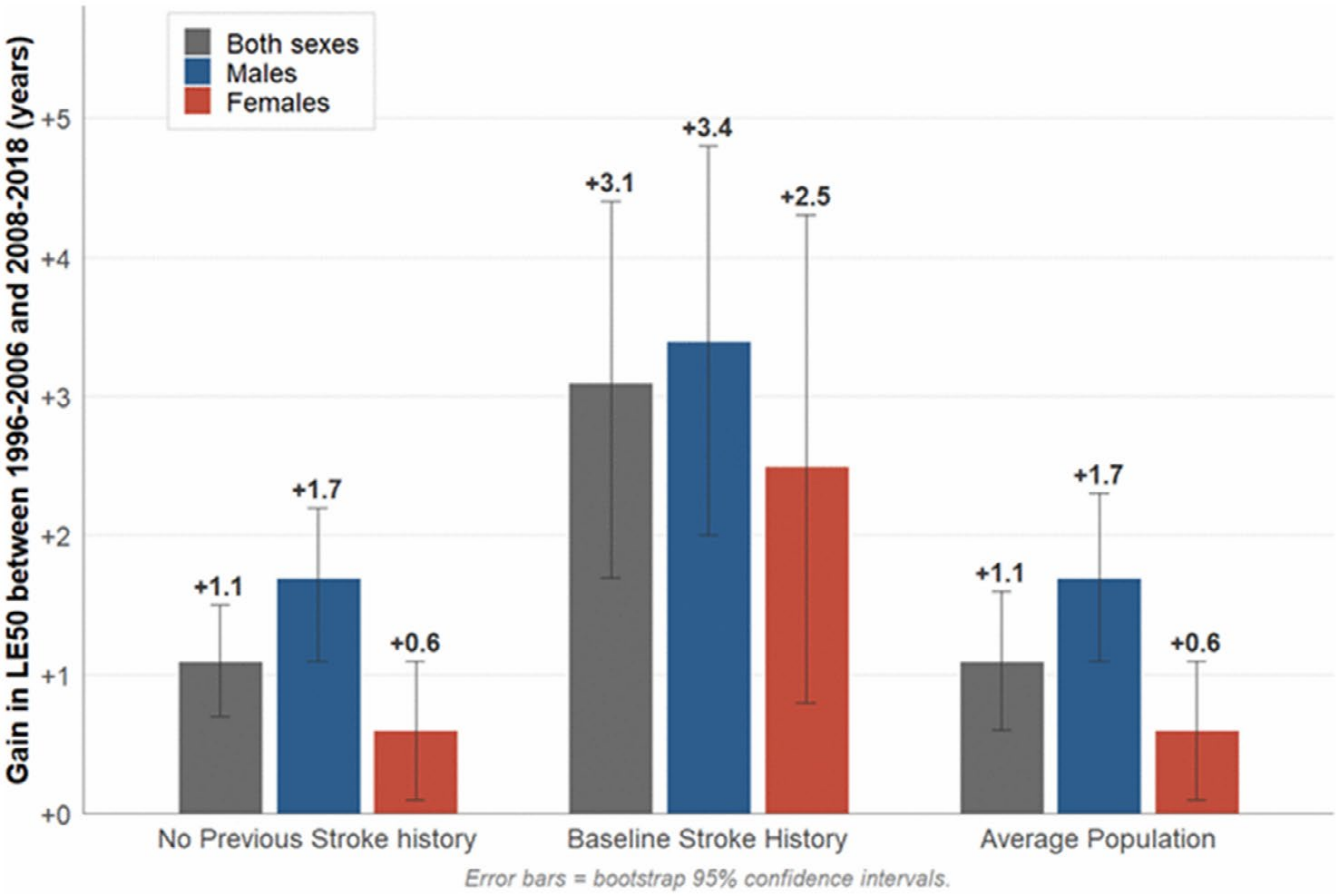
Changes in life expectancy at Age 50 (LE50) between 1996–2006 and 2008–2018, by starting state and sex. Error bars indicate bootstrap 95% confidence intervals derived from independent period‐specific bootstraps.

LE50 estimates by period, sex, and starting state are reported in Table 4, with Table S7 showing transition probabilities and Table S8 the gains and 95% CIs for changes over time in Figure 1.

**TABLE 4:** Life expectancy at Age 50 by period, sex, and starting state.

Starting state		Lifetime with stroke (years)			Lifetime with stroke (years)	
	Lifetime without stroke (years)	1996–2006	Total life expectancy (years)	Lifetime without stroke (years)	2008–2018	Total life expectancy (years)
Both sexes						
No stroke start	27.1 (26.8–27.4)	2.2 (2.1–2.34)	29.3 (29.0–29.6)	28.1 (27.8–28.3)	2.3 (2.2–2.6)	30.4 (30.1–30.7)
Stroke history	~0	17.5 (16.6–18.6)	17.5 (16.6–18.6)	~0	20.6 (19.7–21.7)	20.6 (19.7–21.7)
Average population	26.5 (26.1–26.8)	2.6 (2.4–2.77)	29.0 (28.6–29.3)	27.2 (26.9–27.7)	2.9 (2.6–3.1)	30.1 (29.8–30.5)
Males						
No stroke start	25.1 (24.7–25.5)	2.0 (1.83–2.2)	27.1 (26.8–27.5)	26.5 (26.2–27.1)	2.2 (2.1–2.4)	28.8 (28.3–29.2)
Stroke history	~0	16.5 (15.4–17.4)	16.5 (15.4–17.4)	~0	19.9 (18.9–20.9)	19.9 (18.9–20.9)
Average population	24.3 (23.8–24.9)	2.5 (2.1–2.7)	26.8 (26.3–27.2)	25.6 (25.1–26.3)	2.9 (2.4–3.2)	28.5 (28.1–28.9)
Females						
No stroke start	28.8 (28.5–29.2)	2.34 (2.2–2.6)	31.2 (30.9–31.6)	29.3 (28.9–29.6)	2.4 (2.3–2.6)	31.8 (31.5–32.1)
Stroke history	~0	18.9 (17.8–20.4)	18.9 (17.8–20.4)	~0	21.4 (20.4–22.7)	21.4 (20.4–22.7)
Average population	28.2 (27.7–28.7)	2.7 (2.4–3.0)	30.9 (30.6–31.3)	28.5 (28.0–28.9)	3.0 (2.7–3.4)	31.5 (31.1–31.8)

*Note:* From discrete‐time multistate models (dtms). Bootstrap 95% CI.

In both periods, individuals with a baseline stroke history at Age 50 had substantially shorter remaining life expectancy than those without, with shortfalls ranging from approximately 9 to 12 years depending on sex and period. Male stroke survivors had an LE50 of 16.54 years (95% CI: 15.43–17.43) in 1996–2006 and 19.92 years (18.89–20.87) in 2008–2018. Female stroke survivors had an LE50 of 18.92 years (17.82–20.38) and 21.44 years (20.37–22.72), respectively. In 1996–2006, bootstrap CIs for males and females did not overlap, providing evidence of a meaningful sex difference in post‐stroke remaining life expectancy for those with a previous baseline stroke. In 2008–2018, this gap narrowed and CIs overlapped.

For individuals starting stroke‐free at Age 50, incident stroke accounted for a small share of total remaining life expectancy. In 2008–2018, of the 30.4 total expected years, approximately 28 were expected to be lived without stroke, with the remaining years (ranging between 2.03 and 2.44 depending on sex and period) reflecting the population‐average contribution of incident strokes, driven by the minority of individuals who will experience a stroke during their remaining lifetime. This proportion remained stable across periods and sexes. For individuals already living with stroke history at baseline, all remaining years were spent in the stroke state, as per model specification, where gains in life expectancy were observed.

LE50 improved between periods for all groups, but gains were substantially larger for individuals with stroke history at baseline than for the average population (Figure 1 and Table S8). Since the two periods were bootstrapped independently, CIs for gains were derived by combining the standard errors from each period (the standard error of the gain is the square root of the sum of the squared standard errors from the 1996–2006 and 2008–2018 bootstraps, where each period‐specific standard error was recovered from its bootstrap CI). For both sexes combined, individuals with stroke history gained 3.05 years of LE50 (95% CI: 1.66–4.44), compared to 1.10 years (0.61–1.59) for the average population. Male individuals with stroke history gained 3.38 years (1.97–4.79), compared to 1.66 years (1.06–2.26) for the male average population. Female individuals with stroke history gained 2.52 years (0.78–4.26), compared to 0.58 years (0.07–1.09) for the female average population. Despite the apparent sex difference in absolute gains (males +3.38 vs. females +2.52 years among those with stroke history), CIs for these gains overlapped substantially, indicating that the difference in improvement between sexes remains uncertain.

This was corroborated by two sensitivity analyses: we randomly assigned each overlapping individual to a single period (with equal probability) and re‐estimated all Cox models on this restricted sample where no individual appears in more than one period. This can be found in Table S9, confirming similar coefficients as in the original specification and that results are not driven by the overlapping individuals in the sample. Additionally, we performed a pooled Cox model to obtain a stroke‐period‐sex interaction (available in Table S10), confirming that this female advantage reflects the general sex difference in mortality (female HR = 0.73, *p* < 0.001) rather than a stroke‐specific effect interaction.

## 4 Discussion

Life expectancy at Age 50 among individuals with baseline stroke history improved substantially between 1996–2006 and 2008–2018, gaining approximately 3 years of life (from 17.54 to 20.59 years for both sexes combined) compared to approximately 1 year (from 29.02 to 30.12 years) for the general population. Despite this narrowing, stroke continues to have severe life‐shortening effects: a gap of approximately 10 years persisted in 2008–2018 between individuals with a previous baseline stroke and the general population, representing a relative gap of approximately 30% (a decrease from a gap of approximately 40% in 1996–2006). These relative improvements are consistent with findings from Sweden, where individuals with stroke history also experienced proportionally larger LE gains than the general population [39]. Point estimates suggested larger gains among men (+3.38 years) than women (+2.52 years), though CIs overlapped and we cannot confirm a difference between sexes. The larger absolute gains among stroke survivors relative to the general population, and among males relative to females, may partly reflect diminishing returns: populations with a lower baseline life expectancy have greater capacity for absolute improvement from the same advances in care, compared to those already closer to the overall survival frontier. A similar logic applies to the larger gains observed among male stroke survivors (baseline 16.54 years) compared to female stroke survivors (baseline 18.92 years).

Women with stroke history nevertheless lived longer than men, in 1996–2006 (males: 15.43–17.43; females: 17.82–20.38) that converged in 2008–2018 as male stroke survivors closed part of the gap and CIs overlapped (meaning that it is still possible that women with a baseline stroke still live longer than men). While some population‐based studies have reported higher crude post‐stroke mortality in women [40], this is largely driven by women experiencing strokes at older ages. When comparing at the same age, as the DTMS model does by conditioning on Age 50, women with stroke history lived longer in the 1996–2006 period, consistent with findings from other studies and meta‐analyses [41, 42]. Exploratory Cox models for all‐cause mortality confirmed that the female advantage reflects the general sex difference in survival rather than a stroke‐specific protective effect (Table S11).

Several factors may contribute to the observed improvements in post‐stroke survival. A previous study found that survival following ischemic stroke in the Rotterdam Study improved substantially between 1991 and 2015 while hemorrhagic stroke survival remained unchanged, suggesting that gains are driven primarily by ischemic stroke management [16]. Advances in acute stroke treatment, including the expansion of intravenous thrombolysis with tissue plasminogen activator (tPA) and the adoption of mechanical thrombectomy for large vessel occlusions after 2015, have improved early survival [19]. In parallel, improvements in secondary prevention (including wider use of statins, antihypertensives, and anticoagulation therapy) and the proliferation of organized stroke unit care have likely contributed to longer‐term survival gains [43]. These treatment advances coincide with our observation that gains were concentrated among those with prevalent stroke history rather than among those experiencing incident strokes during follow‐up. Time spent living after an incident stroke did not show a meaningful increase across periods (nor changes in the hazard ratio in the exploratory Cox models), consistent with European findings where stroke mortality declined while incidence remained stable [44,45]. However, the limited number of incident strokes observed in the HRS may reduce our power to detect changes in outcomes of acute strokes.

Gains in total LE50 for the average population reflected contributions from both increased stroke‐free years and longer survival with stroke. Among males, gains were primarily driven by stroke‐free years (+1.25 of 1.66 total), while among females both components showed only marginal improvement (+0.31 stroke‐free, +0.27 with stroke), consistent with stagnating female cardiovascular health gains in the United States [15]. Among covariates, overweight categories were associated with lower mortality. This association, consistent with the obesity paradox in older adults, persisted after simultaneously controlling for conditions commonly linked to excess weight, such as diabetes, hypertension, and heart disease, which independently increased mortality risk [36]. Multicollinearity diagnostics confirmed that BMI and these metabolic conditions captured distinct aspects of health status (all GVIF below 1.25; Table S5).

This study has a series of limitations that we openly acknowledge. First, stroke status in the HRS is self‐reported, which may introduce ascertainment bias. Moreover, the HRS records whether a stroke has ever occurred but not the age or date of the original event, preventing us from distinguishing strokes that occurred recently before study entry from those that may have occurred decades earlier. Second, the HRS does not capture stroke type (ischemic vs. hemorrhagic), stroke severity, or the number of stroke events. Third, while we control for self‐reported heart disease, which captures cardiac comorbidity broadly including atrial fibrillation, we cannot isolate atrial fibrillation as a distinct covariate because more specific questions about heart rhythm disorders are not consistently available across all HRS waves in the RAND harmonized file. Data on medication use and healthcare utilization are also limited, preventing direct examination of treatment effects. Fourth, a total of 17,334 individuals contributed observations to both periods. This overlap is inherent to the longitudinal design of the HRS and is common in studies estimating multistate life expectancy trends from panel data [32, 33]. However, as Table S8 confirms, results are not driven by this overlap since after random assignment results are still substantial. Fifth, the use of BMI as a categorical covariate is limited by the well‐known shortcomings of BMI as a measure of body composition, particularly in older adults where age‐related changes in height and muscle mass may affect classification.

Our findings pertain to stroke survivors observed in an older adult population (among those with a previous stroke history mean age at baseline was approximately 70 years) and should not be extrapolated to young‐onset stroke, which has distinct biological, etiological, and prognostic features that require dedicated study [46]. We believe that despite these limitations, this study provides the first multistate life expectancy estimates for stroke survivors in the United States across two distinct periods at a population level after Age 50, drawing on over 200,000 person‐observations from the HRS. Our findings carry a clear message: while stroke survivors gained approximately 3 years of life expectancy between the late 1990s and the late 2010s, highlighting improvements in long‐term post‐stroke survival, a 10‐year gap between those with and without stroke history persists. Continued investment in both acute stroke care and long‐term secondary prevention remains essential to further close this gap. Future research using clinical registries with stroke type, severity, and treatment data could help identify which specific advances have driven these improvements, and whether they have been equitably distributed across sex and socioeconomic groups.

## Author Contributions

**Octavio Bramajo:** conceptualization, methodology, software, validation, formal analysis, investigation, data curation, writing – original draft, writing – review and editing, visualization, project administration. **Moumita Chakraborty:** methodology, investigation, software, writing – review and editing, supervision. **Lynda Lisabeth:** methodology, investigation, writing – review and editing, supervision. **Neil Mehta:** conceptualization, methodology, investigation, resources, writing – review and editing, supervision, project administration, funding acquisition.

## Funding

This work was supported by National Institute on Aging, R01AG075208.

## Disclosure

The National Institute on Aging (R01AG075208, PI: N.M.) provided funding for this investigation, but did not have any role in the design, conduct, or reporting of this study. The contents of this article are the sole responsibility of the authors. O.B., M.C., and L.L. do not report any financial disclosures. Some parts of this manuscript were edited by Claude AI (Anthropic, San Francisco, CA). The final content and edits are the sole responsibility of the authors.

## Conflicts of Interest

The authors declare no conflicts of interest.

## Supporting information


**Material S1:** Variable definitions for the analysis.
**Figure S1:** Starting (S1 and S2), transition (T) and absorbing (A) states for the multistate models.
**Table S1:** New entrants to the HRS sample by wave, with mean age at first interview.
**Table S2:** Birth‐cohort composition of age‐50 starting observations, by analysis period.
**Table S3:** Missing data.
**Table S4:** Age‐specific mortality rates per 1000 person‐waves, by race/ethnicity (*n* = observations).
**Table S5:** Age composition by race/ethnicity and period.
**Table S6:** Schoenfeld proportional hazards test.
**Table S7:** Average transition probabilities from the discrete‐time multistate models.
**Table S8:** Changes in life expectancy at Age 50 with 95% confidence intervals, by starting state and sex.
**Table S9:** Cox sensitivity analysis—random period assignment.
**Table S10:** Pooled cox models with sex interactions.
**Table S11:** Generalized variance inflation factors (GVIF) from Cox Models 1 and 3.
