# Supplementary material for: Changes in Life Expectancy After Stroke Among Americans Aged 50 and Above by Sex, 1996–2018
Source: J Am Geriatr Soc. 2026 Jul 11;74(9):2583–94. doi: 10.1111/jgs.70585 (PMC13589667; doi:10.1111/jgs.70585)
Supplement: Supplementary file 1 [file JGS-74-2583-s001.pdf]

Supplementary Material

Supplementary Material S1. Variable Definitions for analysis

Stroke variables:

- **Stroke baseline:** Self-reported history of stroke at the first wave of each period ("Has a doctor ever told you that you had a stroke?"). Binary: 0 = No, 1 = Yes.
- **Incident stroke (Incident Stroke):** New stroke reported during follow-up among those stroke-free at baseline. Includes both first-ever strokes and recurrent strokes. Binary, time-varying.

Demographic variables:

- **Age:** Continuous, time-varying. Calculated as interview year minus birth year, adjusted for birth month. Used as the timescale in survival models. Considered a two-year step variable in DTMS life expectancy models.
- **Sex:** RAND variable `ragender`. Male = 1, Female = 2. Models are sex-stratified.
- **Race/ethnicity:** Constructed from RAND variables `raracem` (race) and `rahispan` (Hispanic indicator). Categories: Non-Hispanic White (raracem = 1, rahispan = 0), Non-Hispanic Black (raracem = 2, rahispan = 0), Hispanic (rahispan = 1, any race), Non-Hispanic Other (raracem = 3, , rahispan = 0). Reference: Non-Hispanic White.

Socioeconomic variables:

- **Education:** RAND variable `raeduc` (5-level). Recoded to three categories: Low (raeduc 1-2: less than high school or GED), Medium (raeduc 3-4: high school graduate or some college), High (raeduc 5: college degree and above). Reference: Low.
- **Insurance type:** Constructed from RAND variables `higov` (government insurance indicator) and `prpcnt` (number of private insurance plans). Categories: Private only (no government insurance, at least one private plan), Government only (government insurance, no private plans), Both (government insurance and at least one private plan), Uninsured (no government or private insurance). Reference: Private only.

Health risk factors:

- **Ever smoked:** RAND variable `smokev`. Binary: 0 = Never smoked, 1 = Ever smoked.
- **Hypertension:** RAND variable `hibpe` (ever diagnosed with high blood pressure). Binary: 0 = No, 1 = Yes.
- **Diabetes:** RAND variable `diabe` (ever diagnosed with diabetes). Binary: 0 = No, 1 = Yes.
- **Heart disease:** RAND variable `heart` (ever told by a doctor that you have heart problems). Binary: 0 = No, 1 = Yes. This variable captures cardiac comorbidity broadly, given that other specific heart conditions cannot be isolated in the RAND harmonized file to the best of our knowledge.
- **BMI:** RAND variable `bmi`, derived from self-reported height and weight. Due to differences in coding across HRS waves, raw values were harmonized: values >100 were divided by 10 (later waves stored BMI × 10), while values ≤100 were retained as-is (earlier waves stored BMI in the

original scale). Categorized as: Underweight (<18.5 kg/m²), Normal (18.5-24.9 kg/m²), Overweight (25.0-29.9 kg/m²), Obese (≥30.0 kg/m²). Reference: Normal.

Period variable:

- **Period:** Indicator for observation period. 0 = 1996-2006 (HRS waves 3-8), 1 = 2008-2018 (HRS waves 9-14).

Note: All variables are from the RAND HRS Longitudinal File (v2024). Health measures are self-reported at each wave.

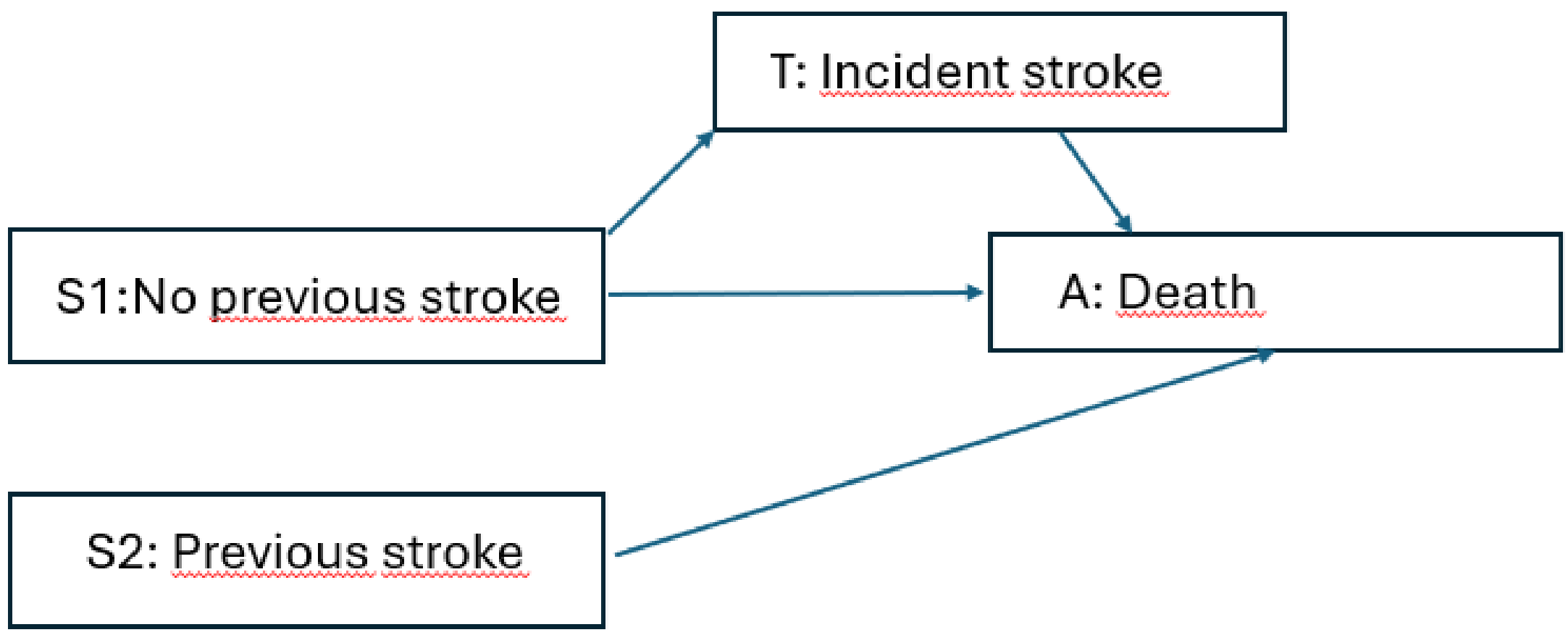

Supplementary Figure S1: Starting (S1 and S2), transition (T) and absorbing (A) states for the multistate models.

Supplementary Table S1. New entrants to the HRS sample by wave, and mean age at first interview.

Wave (year)	New entrants (N)	Age range at entry	Birth cohort(s) entering
1994	18,846	50 to 104	HRS original cohort (1931-41) + AHEAD (before 1923)
1996	836	50 to 94	spouses and late entrants
1998	4,953	50 to 90	Refreshment, born 1924-30 and 1942-47
2000	525	50 to 100	spouses and late entrants
2002	387	50 to 88	spouses and late entrants
2004	3,089	50 to 88	Refreshment, born 1948-53
2006	439	50 to 93	spouses and late entrants
2008	302	50 to 92	spouses and late entrants
2010	5,797	50 to 91	Refreshment, born 1954-59
2012	598	50 to 88	spouses and late entrants
2014	395	50 to 88	spouses and late entrants
2016	4,155	50 to 87	Refreshment, born 1960-65
2018	373	50 to 87	spouses and late entrants
2020	307	50 to 92	spouses and late entrants

Note. Entry wave is defined as the first wave in which the respondent completed an interview (iwstat = 1 in the HRS RAND File). The large influxes at 1998, 2004, 2010, and 2016 correspond to the HRS steady-state refreshment design, which enrolls a new nationally representative sample of adults aged approximately 51-56 every six years (Sonnega et al., 2014). The 1994 row captures the original HRS cohort and AHEAD cohort (waves 2-3 of HRS, fielded 1992-1994). Entries in non-refreshment waves consist primarily of spouses of existing respondents and occasional late additions.

Supplementary Table S2. Birth-cohort composition of age-50 starting observations, by analysis period.

Birth cohort	1996-2006 N	1996-2006 %	2008-2018 N	2008-2018 %
Born 1942-1947	1,344	39.8	0	0.0
Born 1948-1953	1,712	50.7	0	0.0
Born 1954-1959	321	9.5	1,858	43.6
Born 1960-1965	0	0.0	2,115	49.6
Born 1966+	0	0.0	289	6.8
**Total**	**3,377**	**100.0**	**4,262**	**100.0**

Note. Age-50 starting observations are defined as observations with age between 50 and 51.9 years that occur at a wave within the corresponding analysis period (1996-2006 or 2008-2018). Column percentages may not sum to 100.0 due to rounding.

Supplementary Table S3: Missing Data

Models 1–2 include stroke, period, age, education, smoking, hypertension, and diabetes. Models 3–4 additionally include heart disease, BMI, insurance type, and race/ethnicity. Complete-case analysis used throughout.

	Males	Females
Panel A: Analytic sample		
**Total observations**	94,654	130,662
**Total individuals**	17,209	21,875
Models 1–2 (observations)	93,755 (99.0%)	129,763 (99.3%)
Models 3–4 (observations)	93,123 (98.4%)	126,768 (97.0%)
Panel B: Missing observations (Models 1–2 covariates)		
Stroke baseline	39 (0.04%)	51 (0.04%)
Education	29 (0.03%)	12 (0.01%)
Smoking history	847 (0.89%)	839 (0.64%)
Panel C: Missing observations (additional Models 3–4 covariates)		
Heart disease	70 (0.07%)	128 (0.10%)
BMI category	537 (0.57%)	2,914 (2.2%)
Race/ethnicity	60 (0.06%)	21 (0.02%)
Panel D: Individuals with any missing value		

Note: Other variables (Period, Age, Sex, Hypertension, Diabetes, Insurance Type) had no missing cases

Supplementary Table S4: Age-Specific Mortality Rates (per 1,000) by Race/Ethnicity (n= observations)

Age group	NH-White	NH-Black	Hispanic	NH-Other

**50–60**	1.3 (n=38,975)	2.1 (n=13,321)	1.0 (n=9,613)	1.2 (n=2,840)
**60–70**	2.9 (n=47,577)	4.2 (n=12,246)	2.2 (n=7,921)	3.2 (n=1,791)
**70–80**	7.5 (n=42,393)	9.4 (n=7,379)	6.1 (n=4,562)	5.4 (n=1,088)
**80–90**	21.5 (n=22,852)	23.8 (n=3,304)	19.6 (n=1,860)	23.3 (n=488)
**90+**	65.8 (n=4,271)	60.7 (n=634)	48.4 (n=358)	38.9 (n=54)

Supplementary Table S5: Age Composition by Race/Ethnicity and Period

Period	NH-White, Mean Age (SD)	NH-Black, , Mean Age (SD)	Hispanic, , Mean Age (SD)	NH-Other , Mean Age (SD)
**1996–2006**	68.2 (10.6)	66.7 (10.4)	65.7 (10.1)	65.3 (10.1)
N individuals	20,478	4,000	2,344	619
% aged 75+	27.6%	21.9%	19.3%	19.1%
**2008–2018**	69.4 (11.1)	64.5 (10.2)	63.8 (10.0)	62.6 (10.0)
N individuals	17,814	5,789	4,115	1,229
% aged 75+	32.8%	17.8%	16.0%	14.8%

Supplementary Table S6: Schoenfeld Proportional Hazards Test

*P-values from the Schoenfeld residuals test. Significance: *** p<.001, ** p<.01, * p<.05.*

	Males		Females	
**Variable**	**Model 3**	**Model 4**	**Model 3**	**Model 4**
Main exposure variables				
Baseline stroke	0.256	0.265	0.464	0.466
Incident stroke	0.819	0.825	0.091	0.098
Demographics and health covariates				
Period	<.001***	<.001***	<.001***	<.001***
Age	0.201	0.198	0.113	0.120
Education	0.856	0.866	0.981	0.982
Smoking	0.730	0.731	0.915	0.900
Hypertension	0.364	0.356	0.071	0.069
Diabetes	0.215	0.213	0.459	0.484
Heart disease	0.419	0.426	<.001***	<.001***
BMI category	<.001***	<.001***	0.011*	0.011*
Insurance type	0.661	0.659	0.002**	0.002**
Race/ethnicity	0.261	0.257	0.019*	0.017*
Period * Covariate interactions				
Period * Baseline stroke	0.014*	0.014*	0.131	0.137
Period * Incident Stroke	0.017*	0.018*	0.966	0.964
Period * Smoking	—	<.001***	—	<.001***
Period * Education	—	<.001***	—	<.001***
Period * Hypertension	—	<.001***	—	<.001***
Period * Diabetes	—	<.001***	—	<.001***
Period * Heart disease	—	<.001***	—	<.001***
Period * BMI category	—	<.001***	—	<.001***
Period * Insurance type	—	<.001***	—	<.001***
Period * Race/ethnicity	—	<.001***	—	<.001***

Note: The main exposure variables (baseline stroke and incident stroke) satisfy the proportional hazards assumption across all model specifications for both sexes (all p>.05). Stroke * period interactions show marginal violations in males (p=.014–0.018) but not females (p>.09). Violations are concentrated in the period variable (p<.001 in all models), which is expected given that it captures secular mortality trends over a decade.

Supplementary Table S7: Average Transition Probabilities of state from Discrete-Time Multistate Models

	1996–2006			2008–2018		
**From state → To state**	**No Stroke**	**Stroke**	**Death**	**No Stroke**	**Stroke**	**Death**
Both Sexes						
**No previous Stroke**	0.92	0.02	0.06	0.93	0.02	0.05
**Stroke History**	0	0.83	0.17	0	0.85	0.15
Males						
**No previous Stroke**	0.91	0.02	0.07	0.93	0.02	0.05
**Stroke History**	0	0.83	0.17	0	0.84	0.16
Females						
**No previous Stroke**	0.93	0.02	0.05	0.94	0.01	0.05
**Stroke History**	0	0.83	0.17	0	0.85	0.15

Note : *Average biennial transition probabilities across all ages (50–108), by period and sex. Stroke is treated as an absorbing transient state (no recovery to stroke-free). Transition probabilities are estimated via multinomial logistic regression with age as the sole predictor.* The transition from Stroke History to No Stroke is set to 0 by design, reflecting the irreversible nature of stroke history in this model. These transition probabilities feed into the Markov chain calculations that produce the life expectancy estimates in Table 3.

Supplementary Table S8: Changes in Life Expectancy at Age 50 with 95% Confidence Intervals, by Starting State and Sex

From discrete-time multistate models. Period-specific 95% CIs from block bootstrap. Gain CIs derived from independent period-specific bootstraps (SE of difference = $\sqrt{SE_1^2 + SE_2^2}$). Relative gain = gain / baseline value. Ratio CIs computed as lower bound of numerator / upper bound of denominator to upper bound of numerator / lower bound of denominator. Total LE50 (years).

Both sexes				
**Starting state**	**1996–2006 (95% CI)**	**2008–2018 (95% CI)**	**Gain (95% CI)**	**Relative gain (95% CI)**
**No Stroke start**	29.3 (29.0–29.6)	30.4 (30.1–30.7)	**+1.1 (0.7 to 1.5)**	+3.8% (+2.4% to +5.2%)
**Stroke History**	17.5 (16.6–18.6)	20.6 (19.7–21.7)	**+3.1 (1.7 to 4.4)**	+17.4% (+9.5% to +25.3%)
**Average population**	29.0 (28.6–29.3)	30.1 (29.8–30.5)	**+1.1 (0.6 to 1.6)**	+3.8% (+2.1% to +5.5%)
Males				
**Starting state**	**1996–2006 (95% CI)**	**2008–2018 (95% CI)**	**Gain (95% CI)**	**Relative gain (95% CI)**
**No Stroke start**	27.1 (26.8–27.5)	28.8 (28.3–29.2)	**+1.7 (1.1 to 2.2)**	+6.2% (+4.1% to +8.3%)
**Stroke History**	16.5 (15.4–17.4)	19.9 (18.9–20.9)	**+3.4 (2.0 to 4.8)**	+20.4% (+11.9% to +29.0%)
**Average population**	26.8 (26.3–27.2)	28.5 (28.1–28.9)	**+1.7 (1.06 to 2.3)**	+6.2% (+4.0% to +8.4%)
Females				
**Starting state**	**1996–2006 (95% CI)**	**2008–2018 (95% CI)**	**Gain (95% CI)**	**Relative gain (95% CI)**

**No Stroke start**	31.2 (30.9–31.6)	31.8 (31.5–32.1)	**+0.6 (0.1 to 1.1)**	+2.0% (+0.4% to +3.5%)
**Stroke History**	18.9 (17.8–20.4)	21.4 (20.4–22.7)	**+2.5 (0.8 to 4.3)**	+13.3% (+4.1% to +22.5%)
**Average population**	30.9 (30.6–31.3)	31.5 (31.1–31.8)	**+0.6 (0.1 to 1.1)**	+1.9% (+0.2% to +3.5%)

Baseline Stroke survivor gain relative to average population gain			
**Group**	**Stroke gain (95% CI)**	**Avg pop gain (95% CI)**	**Ratio (95% CI)**
**Both sexes**	+3.1 (1.66–4.44)	+1.1 (0.61–1.59)	**2.77× (1.04× to 7.28×)**
**Males**	+3.4 (1.97–4.79)	+1.7 (1.06–2.3)	**2.04× (0.87× to 4.52×)**
**Females**	+2.5 (0.78–4.26)	+0.6 (0.07–1.09)	**4.34× (0.72× to 60.9×)**

Supplementary Table S9: Cox Sensitivity Analysis — Random Period Assignment

*17,334 overlapping individuals randomly assigned to one period only (seed=12345). 1996–2006: 18,736 individuals; 2008–2018: 20,348 individuals. Hazard ratios (95% CIs) shown for stroke-related variables only; all other covariates included as in main analysis Based on Model 1 and Model 3 *** p<.001, * p<.05.*

	Males		Females	
**Variable**	**Main**	**Sensitivity**	**Main**	**Sensitivity**
Model 1				
Baseline stroke	1.70 (1.54–1.88)***	1.70 (1.53–1.89)***	1.83 (1.66–2.0)***	1.70 (1.54–1.88)***
Incident stroke	1.58 (1.39–1.80)***	1.54 (1.32–1.79)***	1.79 (1.59–2.01)***	1.84 (1.61–2.10)***
Baseline stroke * Period	1.03 (0.89–1.19)	1.07 (0.90–1.27)	0.93 (0.81–1.07)	1.18 (1.00–1.40)*
Incident stroke * Period	1.04 (0.83–1.31)	1.26 (0.97–1.66)	1.05 (0.84–1.30)	1.24 (0.99–1.57)
Concordance	0.774	0.774	0.803	0.802
Model 3 (adjusted)				
Baseline stroke	1.67 (1.51–1.86)***	1.50 (1.35–1.67)***	1.82 (1.64–2.02)***	1.60 (1.44–1.77)***
Incident stroke	1.56 (1.37–1.78)***	1.38 (1.18–1.62)***	1.70 (1.51–1.91)***	1.69 (1.48–1.93)***
Baseline stroke * Period	0.97 (0.83–1.13)	1.10 (0.92–1.31)	0.93 (0.81–1.07)	1.12 (0.95–1.33)
Incident stroke *Period	0.96 (0.76–1.22)	1.32 (1.00–1.73)*	1.04 (0.83–1.30)	1.25 (0.99–1.58)
Concordance	0.793	0.790	0.819	0.817

Note: *17,334 overlapping individuals randomly assigned to one period only (seed=12345). 1996–2006: 18,736 individuals; 2008–2018: 20,348 individuals. Hazard ratios (95% CIs) shown for stroke-related variables only; all other covariates included as in main analysis. *** p<.001, * p<.05.*

Supplementary Table S10: Pooled Cox Models with Sex Interactions

*Hazard ratios (95% CIs) from pooled models combining males and females. Model A tests whether the stroke mortality penalty differs by sex. Model B additionally tests whether the change in stroke mortality across periods differs by sex (three-way interaction). Both models adjust for education, smoking, hypertension, diabetes, heart disease, BMI, insurance type, and race/ethnicity. *** p<.001, * p<.05. — = not included in model.*

Variable	Model A	Model B
Main effects		
Baseline stroke	1.67 (1.53–1.82)***	1.69 (1.54–1.86)***
Incident stroke	1.53 (1.37–1.72)***	1.55 (1.37–1.76)***
Female (ref: Male)	0.73 (0.70–0.76)***	0.72 (0.68–0.76)***
Period 2008–2018	0.58 (0.55–0.61)***	0.56 (0.53–0.60)***
Age (per year)	1.07 (1.07–1.07)***	1.07 (1.07–1.07)***
Stroke * Period interactions		
Baseline stroke * Period	0.96 (0.86–1.07)	1.00 (0.87–1.15)
Incident stroke * Period	1.01 (0.85–1.21)	1.10 (0.87–1.39)
Stroke * Sex interactions		

Baseline stroke * Female	1.07 (0.96–1.18)	1.12 (0.98–1.28)
Incident stroke * Female	1.19 (1.02–1.39)*	1.20 (0.98–1.48)
Sex * Period interaction		
Female * Period	—	1.05 (0.97–1.14)
Stroke * Sex * Period interactions		
Baseline stroke * Female * Period	—	0.90 (0.73–1.11)
Incident stroke * Female * Period	—	0.97 (0.69–1.35)
Model fit		
N (observations)	219,891	219,891
AIC	281,060	281,057
Concordance	0.805	0.805
LR test (B vs A)		p = .071

Note: The female main effect (HR=0.73, p<.001) confirms a general female survival advantage. The baseline stroke × female interaction is not significant (HR=1.07, p=.21), indicating that stroke penalizes both sexes equally. The three-way interactions are not significant (baseline stroke × female × period: HR=0.90, p=.32; incident stroke × female × period: HR=0.97, p=.85), indicating that gains between periods were not different by sex. The LR test (p=.071) confirms that the three-way terms do not improve fit.

Supplementary Table S11: Generalized Variance Inflation Factors (GVIF) from Cox Models 1 and 3

Variable	GVIF^(1/2df) Males	GVIF^(1/2df) Females
Baseline stroke	1.02	1.02
Incident stroke	1.01	1.01
Period	1.06	1.05
Age	1.19	1.22
Education	1.04	1.04
Smoking	1.01	1.03
Hypertension	1.06	1.06
Diabetes	1.05	1.08
Heart disease	1.04	1.04
BMI category	1.03	1.03
Insurance type	1.07	1.06
Race/ethnicity	1.04	1.04

*Note: GVIF^(1/2*df) is the appropriate measure for categorical variables with >1 df. Values below 2.5 indicate no multicollinearity concern.*

The highest pairwise correlation was between diabetes and hypertension (r=0.23). Among obese individuals, 68% did not have diabetes, confirming that BMI and diabetes capture distinct aspects of metabolic risk despite their association. VIF models exclude interaction terms, as VIF is not defined for interaction-only models
